# Editorial: AI-driven advances in personalized nutrition through optimization in food manufacturing

**DOI:** 10.3389/fnut.2026.1956413

**Published:** 2026-09-03

**Authors:** Navneet Kumar, Sudeep Tanwar, Anurag Singh

**Affiliations:** 1 National Academy of Agricultural Research Management (ICAR), Hyderabad, Telangana, India; 2 Marwadi University, Rajkot, Gujarat, India; 3 Harcourt Butler Technical University, Kanpur, Uttar Pradesh, India

**Keywords:** artificial intelligence, food manufacturing, food quality, machine learning, optimization, personalized nutrition, precision nutrition

Artificial intelligence (AI) is combining with nutrition science and food manufacturing to transform how food is created, formulated, and tailored to individual dietary needs. Whereas food production was previously ruled mostly by empirical trial-and-error and broad dietary recommendations, it is becoming progressively dictated by predictive modeling along with real-time optimization and data-driven personalization. Together, these five contributions comprise a comprehensive survey of this transformation across conceptual frameworks, restaurant-level decision support, cereal product optimization and pharmaceutical-grade formulation stability within this Research Topic “*AI-driven advances in personalized nutrition through optimization in food manufacturing*.” They can collectively show that the utility of AI applied in this domain is not through any identifiable algorithm, but rather from the ability to unify heterogeneous data— sensory, biochemical, physiological and operational—into actionable individualized and industrially reproducible solutions.

## 1 Mapping the landscape: reviews and conceptual frameworks

The conceptual scaffolding for the Research Topic is established by two contributions by Agrawal, Goktas, Holtkemper et al.. The integrated review of application of artificial intelligence (AI) in food manufacturing for providing evidence for the use of computer vision, real time monitoring and predictive analytics leverage the key application for workflow streamlining, waste recycling and enabling of circular economy. The systematic study highlights the structural obstruction for the adoption, weakness in infrastructure, ethical concern about the data privacy, and the cost involved in the implementation for small and medium manufacturers, indicating that technological capability is not enough without accompanying governance and financial investment.

On this basis, Agrawal, Goktas, Kumar et al. provide an extensive review that covers both personalized nutrition as well as food manufacturing. Their research tracks the transition away from static, dietary guidelines at population level toward dynamic and personalized frameworks that use multi-omics data harvested by wearable biosensors with reinforcement learning over a patient's lifetime whilst also investigating privacy-preserving approaches including federated learning for compliant management of health-data. The review shapes how ethical and practical scaffolding can meet at the intersection of algorithmic transparency, equitable access and regulatory harmonization alongside technical capability to facilitate the use of AI in a responsible manner connecting healthcare, nutrition and industrial food systems.

## 2 From framework to plate: AI-guided personalized food selection

Seminar et al., inferring preferences about various lifestyle determinants, whether the consumer will prefer a low-sodium option when they are away from sensors, propose a personalized meal selection decision support system based on genetic algorithm in restaurant of Indonesia. The system treats the menu items and their corresponding nutrient profile as chromosomes and encodes each of them before optimizing them using a penalty-based fitness function specific to individual sex, age, weight, height, and non-communicable disease history. It is useful to generate the optimal meal pairings using real-time restaurant data. At a partner restaurant field test site, user-centered evaluation demonstrated enhanced nutritional education and consumer experience, a scalable model for delivering precision nutrition directly in standard food service context, particularly within the often-congested western dietary patterns.

## 3 Optimizing traditional food products with hybrid AI approaches

Türk Aslan et al. show that an integrated Response Surface Methodology (RSM—Particle Swarm Optimization (PSO) approach are useful for the bioactive and sensory properties of Piltaf based three GI Turkish bulgur varieties namely, Siyez, Firik and Karakılçık. The application of AI for finalizing the formulation strategy for the prediction of optimal solution of bulgur to water ratio, which resulted in a study representing the Karakılçık bulgur based pilaf received the highest antioxidant capacity; Firik bulgur pilaf showed the highest flavonoid and total phenolic content and overall acceptability; whilst Siyez bulgur pilaf benefited from having the best taste scores. While the Particle Swarm Optimization validation step confirms the predictive power of Response Surface Methodology models, it also demonstrates how hybrid AI methods may be leveraged to rationalize and improve heritage cereal products for consumer appeal and functional food development, an approach with real-world implications for revolutionizing industrial foods while conserving traditional ones.

## 4 Ensuring safety in complex formulations: predictive stability modeling

Yong-guang et al. conducted a meta-analysis of 1,518 samples from 872 distinct formulations across 19 studies, and trained a cohort of sample descriptors in an XGBoost machine learning model for predicting lipid emulsion stability in parenteral nutrition. This model was predictive (98.2% accuracy and AUC 0.968) and discriminative, with SHAP-based interpretability results revealing amino acid and phosphate concentration, storage time, and lipid composition as the main drivers of stability. This work presents an approach for using AI to overcome cross-laboratory data heterogeneity and provide evidence for clinically actionable tools that make precision nutrition principles applicable in the critical context of personalized parenteral formulations.

## 5 Synthesis and future directions

Taken together, these five contributions form a coherent arc: from high-level reviews synthesizing AI perspectives in food manufacturing and personalized nutrition to consumer facing recommendation networks to product-level formulation level optimization to safety critical stability prediction at the clinical nutrition level. There is a common thread among them—AI's primary contribution lies not simply in automation, but in balancing multiple objectives (taste, nutrition, safety, sustainability and cost), often conflicting, to deliver optimized personalized solutions at scale.

Many challenges that are prevalent across these studies should continue to be targeted: the requirement of larger and representative datasets to enable generalization across populations and products; model interpretability/explainability as a key factor for garnering confidence from clinicians/regulators & end-users in scale-up; resource/infrastructure constraints preventing adoption by smaller producers; finally, equitable utilization of personalized nutrition technologies wherever they may have a role to play. Prospective, multi-site validation studies, further integration of genomic and microbiome data into personalization pipelines, and privacy-preserving architectures for sensitive health and dietary back-end databases are worthy of future work.

We hope that this Research Topic provides a snapshot of progress to date and a basis for scalable, interdisciplinary interaction between data scientists, food technologists, nutritionists and clinicians as the field progresses toward more intelligent, personalized and sustainable food systems.

